# Role of FGF10/FGFR2b Signaling in Homeostasis and Regeneration of Adult Lacrimal Gland and Corneal Epithelium Proliferation

**DOI:** 10.1167/iovs.64.1.21

**Published:** 2023-01-30

**Authors:** Emma N. Finburgh, Olivier Mauduit, Takako Noguchi, Jennifer J. Bu, Anser A. Abbas, Dominic F. Hakim, Saverio Bellusci, Robyn Meech, Helen P. Makarenkova, Natalie A. Afshari

**Affiliations:** 1 Viterbi Family Department of Ophthalmology, Shiley Eye Institute, University of California San Diego, La Jolla, California, United States; 2 Department of Molecular Medicine, The Scripps Research Institute, La Jolla, California, United States; 3 Cardio-Pulmonary Institute and Department of Pulmonary and Critical Care Medicine and Infectious Diseases, Universities of Giessen and Marburg Lung Center, German Center for Lung Research, Justus Liebig University Giessen, Giessen, Germany; 4 Discipline of Clinical Pharmacology, College of Medicine and Public Health, Flinders University, Adelaide, South Australia, Australia

**Keywords:** fibroblast growth factor, FGF10, lacrimal gland, corneal epithelium, dry eye, cell proliferation

## Abstract

**Purpose:**

Fibroblast growth factor 10 (FGF10) is involved in eye, meibomian, and lacrimal gland (LG) development, but its function in adult eye structures remains unknown. This study aimed to characterize the role of FGF10 in homeostasis and regeneration of adult LG and corneal epithelium proliferation.

**Methods:**

Quantitative reverse transcription PCR was used for analysis of FGF10 expression in both early postnatal and adult mouse LG, and RNA sequencing was used to analyze gene expression during LG inflammation. FGF10 was injected into the LG of two mouse models of Sjögren's syndrome and healthy controls. Flow cytometry, BrdU cell proliferation assay, immunostaining, and hematoxylin and eosin staining were used to evaluate the effects of FGF10 injection on inflammation and cell proliferation in vivo. Mouse and human epithelial cell cultures were treated with FGF10 in vitro, and cell viability was assessed using WST-8 and adenosine triphosphate (ATP) quantification assays.

**Results:**

The level of *Fgf10* mRNA expression was lower in adult LG compared to early postnatal LG and was downregulated in chronic inflammation. FGF10 injection into diseased LGs significantly increased cell proliferation and decreased the number of B cells. Mouse and human corneal epithelial cell cultures treated with FGF10 showed significantly higher cell viability and greater cell proliferation.

**Conclusions:**

FGF10 appears to promote regeneration in damaged adult LGs. These findings have therapeutic potential for developing new treatments for dry eye disease targeting the ability of the cornea and LG to regenerate.

The fibroblast growth factors (FGFs) are a family of cell signaling proteins that play an important role in the development and patterning of many tissues, including exocrine glands and eye structures.^1^^,^^2^ Based on their receptor specificity, FGFs are divided into six subfamilies.^3^^–^^7^ The members of the FGF7 subfamily (FGF3, FGF7, FGF10, and FGF22) are usually expressed by mesenchymal cells and signal to “b” isoforms of FGFR1 and FGFR2, expressed on epithelial cells.^3^^,^^8^ FGF10 plays an important role in lens morphogenesis and growth,^9^^–^^11^ lacrimal and meibomian gland induction and branching,^12^^,^^13^ and development of the cornea.^14^ Moreover, FGF10 has been recently implicated in adult tissue homeostasis and stem cell function.^4^^,^^15^^–^^18^ Genetic defects due to mutations in FGF10 signaling can result in a variety of human conditions. The involvement of FGF signaling in ocular disease is well documented. Recent studies have identified mutations in *Fgf10* in people with aplasia of the lacrimal and salivary glands and lacrimo–auriculo–dento–digital syndrome.^19^^–^^21^ Similarly, *Fgfr2* knockout mice have reduced corneal epithelial cell proliferation^14^ and absence of meibomian and lacrimal glands (LGs).^22^^,^^23^ However, the role of FGF10 in adult eye structures and its therapeutic potential have not been well characterized.

One of the most common conditions of the ocular surface is dry eye disease, which is the chronic lack of sufficient lubrication of the ocular surface leading to symptoms of dryness and irritation. Dry eye is a multifactorial disease and can be caused by natural aging, autoimmune diseases, environmental factors such as dry climate, and various medications. The pathogenesis of dry eye syndrome is not fully understood, but it is known to be closely associated with corneal, meibomian gland, and LG function. Recent population-based studies have reported the overall prevalence of dry eye ranging from 21.3% in Canada to 42.0% in Africa, with a larger burden in older age groups.^24^^,^^25^ Altogether, dry eye represents an increasing economic and healthcare burden on an aging global population. Several treatments for dry eye exist, including plugs to reduce tear drainage, artificial tears and gels, and anti-inflammatory drugs such as topical cyclosporine. However, these interventions are palliative and are not aimed at solving the underlying LG deficiency, atrophy, and corneal damage. It has been shown that dry eye syndrome leads to decreased cell proliferation and tissue repair. There is a growing and critical need to develop new comprehensive dry eye disease treatments targeting the ability of the LG and cornea to regenerate.

Several FGF-targeted therapies to treat diseases such as cancer, chronic kidney disease, vascular diseases, and tissue regeneration are already being explored.^26^^–^^28^ To date, several studies have reported a positive effect of FGF10 on epithelial cells. FGF10 upregulates the expression of mucin in conjunctival epithelial cells,^29^ protects the ocular surface in the rabbit dry eye model, and controls epithelial cell migration during embryonic eyelid closure.^30^ Furthermore, a protective role of FGFs during inflammation has been recently reported.^31^ In 2021, Wang et al.^32^ reported that FGF10 significantly reduced the expression levels of the proinflammatory factors IL-1β and TNF-α and improved corneal endothelial cell function. Moreover, patients have been found to develop corneal epithelial changes with decreased visual acuity and dry eye following FGFR inhibitor therapy.^33^^,^^34^ It has been proposed that, in the lung, FGF10 could also impact the immune cell populations^35^; however, the connection between FGF10 and chronic inflammation in eye tissues remains unclear.

In this study, we have characterized the potential of FGF10 as a treatment for dry eye. We tested the in vitro application of FGF10 on primary corneal epithelial and LG cell cultures, as well as in vivo injection of FGF10 into the LGs of two murine models of dry eye disease.

## Materials and Methods

### Mice

Two mouse models of Sjögren's syndrome (SS) were used to assess the generalizability of FGF10 responses in dry eye conditions with different underlying etiologies: thrombospondin-1 null mice (*TSP-1*^−/−^, RRID:IMSR_JAX:006141) and *NOD.B10.H2^b^* (RRID:IMSR_JAX:002591) mice. *TSP-1*^−/−^ mice spontaneously develop multifactorial ocular surface disease that includes LG dysfunction. *TSP-1^−^*^/^*^−^* mice were originally purchased from The Jackson Laboratory (Bar Harbor, ME, USA) and were bred and maintained on the *C57BL/6J* background.

*NOD.B10.H2^b^* mice are a primary SS mouse model, in which the I-A^g7^ segment of the major histocompatibility complex region has been replaced by the H2^b^ haplotype of *C57BL/10SnJ RT* mice. These mice retain the characteristic lymphocytic infiltration and dysfunction of the LGs as seen in humans, but they do not have autoimmune diabetes. *NOD.B10.H2^b^* mice were purchased from The Jackson Laboratory to establish mouse colonies. *BALB/c* mice (RRID:IMSR_JAX:000651) and wild-type (WT) *C57BL/6* mice (RRID:IMSR_JAX:000664) were used as controls for the *NOD.B10.H2^b^* and *TSP-1^−^*^/^*^−^* mice, respectively.

*Rosa26* *rtTA:tet(O)soluble Fgfr2b* (*R2*6*rtTA:tet(O)sFgfr2b*) mice have been described previously.^36^ These mice were crossed with the *Pax6-LacZ* transgenic mouse,^16^ which shows LacZ expression throughout the epithelial component of the LG, to generate the *R26rtTA:tet(O)sFgfr2b:Pax6-LacZ* mice. Inducible attenuation of the FGFR2b pathway in these mice was achieved by administration of doxycycline (DOX)-containing food (rodent diet with 0.0625% DOX; TD.01306; Harlan Teklad Laboratories, Placentia, CA, USA). *Pax6-LacZ* mice were also administered DOX and used as controls. Five mice per each group were used in the experiment. Mice were treated with DOX for 40 to 45 days, and at the end of this period their LGs were injected with IL-1α to induce injury or were injected with vehicle as a control, as described previously.^37^^–^^39^ Five days after LG injury, when main gland regeneration was complete, mice were sacrificed, and the LGs were processed for X-gal staining (see below). This time point was selected based on a previous study showing that healthy LGs regenerate within 5 days after injury.^37^ To evaluate LG regeneration, we analyzed sections made through the central part of each LG, and the percentage of area containing acinar cells was calculated (as percent acini-containing area compared to the whole area of the section).

Mice were housed under standard conditions of temperature and humidity, with a 12-hour light/dark cycle and free access to food and water. All experiments were performed in compliance with the ARVO Statement for the Use of Animals in Ophthalmic and Vision Research and the Guidelines for the Care and Use of Laboratory Animals published by the National Institutes of Health (NIH Publication No. 85-23, revised 1996) and were preapproved by The Scripps Research Institute Animal Care and Use Committee.

### Primers and Quantitative Reverse-Transcription PCR

Total RNA was isolated using the RNeasy Micro Kit (#74004; QIAGEN, Hilden, Germany) and converted into cDNA using RT2 First Strand Kit (#330404, QIAGEN). The quantitative reverse-transcription PCR (qRT-PCR) reactions were performed in a 25-µL PCR total reaction mixture using RT² SYBR Green Fluor qPCR Mastermix (#330513, QIAGEN) according to the manufacturer's instructions and using a 7300 Real-Time PCR System (Applied Biosystems, Waltham, MA, USA). Sequences for two sets of *Fgf10* qRT-PCR primers were (1) forward: 5′-CGG GAC CAA GAA TGA AGA CT-3′, reverse: 5′-GCA ACA ACT CCG ATT TCC AC-3′; and (2) forward: 5′-ATGACTGTTGACATCAGACTCCTT-3′, reverse: 5′-CACTGTTCAGCCTTTTGAGGA-3′. The TaqMan Rodent GAPDH Control Reagent (#4308313; Applied Biosystems) was used as a normalization control. The qRT-PCR data were analyzed using the comparative C_T_ (ΔΔC_T_) method. Prism 9 (GraphPad, San Diego, CA, USA) was used for graphing and statistical analyses. Statistical tests included *t*-tests and one-way ANOVA. *P* < 0.05 was considered significant.

### RNA Sequencing

RNA sequencing data (GSE210332) were used to assess *Fgf10* expression levels in the LG during inflammation and regeneration. In this dataset, LGs from *NOD.B10.H2^b^* and control *BALB/c* mice were sequenced in triplicates at 2, 4, and 6 months of age with a minimal depth of 50 million reads. Three LGs from each group were sequenced. We have recently shown that lymphocytic foci in the LGs of *NOD.B10.H2^b^* males appear around 2 months of age (early disease); by 4 months of age, the LGs develop moderate disease and by 6 months of age severe disease.^40^ The range of 2 to 6 months thus allowed us to study the *Fgf10* expression in the LGs of *NOD.B10.H2^b^* males during the progression of chronic inflammation while avoiding the effect of aging. DEseq2 was used to calculate fold changes and false discovery rate (FDR).

### Flow Cytometry

LGs from *C57BL/6*, *TSP1^–/^^–^*, *BALB/c*, and *NOD.B10.H2^b^* mice were analyzed by flow cytometry. LGs from each mouse were excised and digested in collagenase type IV (#17104019; Thermo Fisher Scientific, Waltham, MA, USA) diluted in Hank's Balanced Saline Solution (HBSS; #H6488; Sigma-Aldrich, St. Louis, MO, USA) for 1 hour at 37°C in a shaking water bath. Cells were collected by centrifugation at 1000 rpm, resuspended in 100-µg/mL DNase (#D4513; Sigma-Aldrich) and 5-mM MgCl_2_ in HBSS, and incubated for 15 to 30 minutes at room temperature. For fluorescence-activated cell sorting (FACS) analysis, approximately 0.5 × 10^6^ cells were pelleted at 1200g for 10 minutes at 4°C and resuspended in 100 µL of staining buffer with the appropriate conjugated antibody: fluorescein isothiocyanate (FITC)-conjugated CD45 mouse monoclonal antibody (clone B-A11, RRID:AB_868944, #ab27287; Abcam, Cambridge, UK) and allophycocyanin (APC)-conjugated CD19 rat monoclonal antibody (Clone eBio1D3 (1D3), RRID:AB_1659676, #17-0193-82; Thermo Fisher Scientific). The cells were stained on ice for 1 hour with gentle vortexing every 15 to 20 minutes, pelleted as above, and resuspended in 1 mL of cold PBE (PBS supplemented with 0.5% BSA and 2 mM EDTA) in FACS tubes. Flow cytometric analysis and FACS were performed at The Scripps Research Institute Flow Cytometry Core Facility using Digital LSR II (BD Biosciences, Franklin Lakes, NJ, USA) and FACS Vantage DiVa (BD Biosciences) instruments. Data analyses were performed using FlowJo software. The main population of cells was determined by forward and side scatter area gating, as well as dead cell exclusion via propidium iodide or 7-aminoactinomycin D. Doublets were excluded via forward scatter area versus width and with side scatter area versus width. Control samples labeled with isotype control antibodies and with a single primary antibody were used to determine the background noise because of nonspecific antibody binding and to establish proper compensation for optimum separation between signals. The percentage of B cell CD45^+^/CD19^+^ was determined using FlowJo software. For each mouse strain, five *Fgf10*-injected LGs and five control LGs (obtained from five mice) were analyzed. Paired *t*-tests were used to determine statistical differences between *Fgf10*- and BSA-injected glands.

### Histology

Extra-orbital LGs from each group (6-month-old *TSP-1^−^*^/^*^−^* and *NOD.B10.H2^b^* mice and corresponding controls) were dissected as we described previously,^41^ fixed in 4% EM grade paraformaldehyde (#00380-1; Polysciences, Inc., Warrington, PA, USA) and embedded in paraffin. Then, 10-µm sections were prepared and stained with hematoxylin and eosin (H&E) to evaluate the morphology.

### Frozen Section Preparation and Immunostaining

To prepare the frozen sections, LGs were fixed with 2% paraformaldehyde in PBS (pH 7.4) for 20 to 30 minutes, frozen in 2-methylbutane (isopentane, #M32631-500ML; Sigma-Aldrich) cooled by liquid nitrogen, and 10-µm sections were prepared using a Bright OTF 5000-LS004 Cryostat (Hacker Instruments, Winnsboro, SC, USA). Sections were blocked with 5% goat serum in Tris-buffered saline containing 0.1% Tween 20 (TBST). The following primary antibody was used for immunostaining: heparan sulfate proteoglycan (clone A7L6 #MAB1948P, RRID:AB_10615958; Sigma-Aldrich).

Appropriate Invitrogen secondary antibodies were obtained from Thermo Fisher Scientific. Images were taken using a Zeiss LSM 780 confocal laser scanning microscope (RRID:SCR_020922; Carl Zeiss Microscopy, Jena, Germany). Isotype-specific immunoglobulins (normal rabbit IgG, #12-370, RRID:AB_145841; normal mouse IgG, #12-371, RRID:AB_145840; Sigma-Aldrich) or pre-immune serum, as a substitute for the primary antibody, were used for negative controls.

### X-Gal Staining

For LacZ detection, 5-bromo-4-chloro-3-indolyl β-D-galactopyranoside (X-Gal, #B4252; Sigma-Aldrich) was dissolved in dimethylformamide 50 mg/mL and stored at −80°C. Stock solutions of 0.5-M K_4_Fe(CN)_6_, 0.5-M K_3_Fe(CN)_6_, 1-M MgCl_2_, 2% NP-40, and 1% sodium deoxycholate were prepared and stored at room temperature. The staining solution was prepared by adding 120 µL of K_3_Fe(CN)_6_, 120 µL of K_4_Fe(CN)_6_, 24 µL of 1-M MgCl_2_, 120 µL of 2% NP-40, 120 µL of 1% sodium deoxycholate, and 240 µL of 50-mg/mL X-Gal, and the volume of staining solution was adjusted by 0.1-M PBS (pH 7.4) up to 12 mL. Fixed tissue was rinsed in 0.1-M PBS (pH 7.4) for 20 to 30 minutes and stained in a X-Gal staining solution at room temperature in the dark. If needed, frozen or paraffin sections were prepared and stained with Fast Red to visualize nuclei.

### Cell Proliferation Assay: BrdU Labeling Index in LG Lobules

To assess the rate of cell proliferation, animals were injected intraperitoneally with bromodeoxyuridine (BrdU, 20 mg/mL in 0.007-M NaOH; Sigma-Aldrich) at a dose of 100 mg/g body mass. Loss of BrdU signal due to competing thymidine incorporation was blocked with 5-fluoro-2′-deoxyuridine (1.4 mg/mL in 0.007-M NaOH; Sigma-Aldrich) at a dose of 6.7 mg/g body mass. Animals were euthanized, and the LGs were harvested and processed for BrdU detection using a BrdU detection kit (#11296736001, RRID:AB_2814711; Roche, Basel, Switzerland). Briefly, LG lobules or sections were permeabilized by Triton X-100 in 0.1-M PBS for 20 to 30 minutes and incubated in 2-N HCl for 30 minutes to denature chromatin. This step was followed by incubation in 0.2-M sodium borate buffer two times for 15 minutes to neutralize acid and washing in a blocking solution of 5% goat serum in 0.1-M PBS (pH 7.4) for 15 minutes. Incubation with primary antibodies was performed overnight at 4°C, followed by washing and incubation with secondary antibodies for 1 hour at room temperature. Quantitation to determine the BrdU labeling index of LG cells was performed using the Zeiss fluorescence microscope. Five randomly selected fields were analyzed per each LG section, and the average percentage of cells incorporating BrdU was determined.

### FGF10 Injection Into the LG

To evaluate the effect of FGF10 on cell proliferation during chronic inflammation, we injected FGF10 (five mice per group; 10 LGs) or BSA (five mice per group; 10 LGs) into the LGs of *NOD.B10.H2^b^* mice, performed cell proliferation assays (see above), and determined focus scores. Four-month-old *NOD.B10.H2^b^* and *TSP-1*^–/–^ mice were anesthetized, and the hair on both sides of each mouse was removed using hair clippers. Skin was disinfected with 10% povidone–iodine solution.^42^ After skin preparation, an approximately 0.5-cm full-thickness skin incision was made in the center of the prepared area using a sterile surgical instrument, and the extra-orbital LG was slowly injected with 2 µL of human recombinant FGF10 (100 ng/µL in PBS) using a 33-gauge needle (#NF33BV-2; World Precision Instruments, Sarasota, FL, USA) and a nanofil syringe (World Precision Instruments). Control LGs were injected with vehicle (PBS). Three injections in each gland into different lobules were performed to ensure larger areas of gland treatment. Analyses of LGs were carried out 3 days after injection.

### Assessment of LG Inflammation by Calculation of Focus Scores

Paraffin sections of LGs stained with H&E were used to calculate the focus scores. The immune foci were defined as mononuclear cell infiltrates containing at least 50 inflammatory cells. The number of foci was divided by the surface area (in mm^2^) of the section and multiplied by 4 to obtain the focus score/4 mm^2^. Three sections per gland were analyzed. The area was measured using ImageJ (National Institutes of Health, Bethesda, MD, USA).

### Isolation of Mouse Primary Corneal Epithelial Cells and FGF10 Application

Mouse eyes were dissected and incubated with dispase (15 mg/mL, neutral protease [Dispase], #44439; Cell Signaling Technology, Danvers, MA, USA) overnight at 4°C. Corneal epithelial cell sheets were gently peeled off corneas and placed into 200 to 500 µL of Gibco Trypsin-EDTA (0.25%), phenol red (#25200072; Thermo Fisher Scientific) at 37°C for 10 to 15 minutes to form single-cell suspensions. Trypsin was neutralized by the addition of one volume (200–500 µL, 10 mg/mL) of MilliporeSigma Cabiochem Trypsin Inhibitor, Soybean (#65035100MG; Thermo Fisher Scientific). Cells were pelleted at 500*g* for 5 minutes and resuspended in defined culture medium (Gibco EpiLife Medium, #MEPI500CA; Thermo Fisher Scientific) supplemented with Gibco Human Corneal Growth Supplement (HCGS, #S0095; Thermo Fisher Scientific), 5 ng/mL Gibco Human EGF Recombinant Protein (EGF, #PHG0313; Thermo Fisher Scientific), Gibco AlbuMAX I Lipid-Rich BSA (#11020; Thermo Fisher Scientific), insulin–transferrin–selenium (ITS-G, 100X, #41400045; Thermo Fisher Scientific), and Gibco Human Transferrin (#0030124SA; Thermo Fisher Scientific). Cells were plated into 65-mm Petri dishes coated with 0.1 mg/mL poly-d-lysine (PDL, #A3890401; Thermo Fisher Scientific) and laminin (EHS sarcoma, mouse, #1243217; Sigma-Aldrich). After 4 days in culture, cells were stripped with Gibco Trypsin–EDTA (0.05%, #25300120; Thermo Fisher Scientific), and replated into 96-well plates coated with PDL/laminin, with 10,000 to 20,000 cells per well in 100 µL of defined culture medium (without growth factors) with or without 25 ng/mL of *Fgf10* in PBS (recombinant human FGF-10, #345-FG; R&D Systems, Minneapolis, MN, USA). Cell growth and viability were tested using the Cell Meter Colorimetric WST-8 Cell Quantification Kit (#22771; AAT Bioquest, Pleasanton, CA, USA) for 1, 3, and 7 days in culture according to the manufacturer’s protocol.

### Plate Coating for Corneal Cell Culture

To prepare plates for the growth of corneal epithelial cells, PDL was added to dishes or wells (65-mm Petri dishes; six-well dishes, 1 mL; 96-well plates, 0.05–0.1 mL) and left for 2 hours or overnight. PDL was then aspirated, and the plates were left for 20 minutes to dry and washed three times in PBS (pH 7.4, #10010023, Thermo Fisher Scientific). The plates were then coated with laminin (#L2020-1MG; Sigma-Aldrich). Laminin stock solution (1 mg/mL) was diluted with PBS. To coat the six-well plates and 65-mm Petri dishes, laminin was diluted with PBS (1:100), and 1 mL was added to each well or dish; to coat the 96-well plates, laminin was diluted with PBS (1:20), and each well was coated with 50 µL of diluted laminin. Plates and dishes with laminin were incubated for 2 hours in a tissue culture incubator at 37°C, laminin was aspirated, and plates were washed three times with PBS prior to cell plating.

### Human Donor Tissue

Human donor corneas were obtained from the San Diego Eye Bank (San Diego, CA, USA). Remaining corneal tissue was collected after trephination or graft preparation for corneal transplant surgery. All corneas had been stored at 4°C in storage medium (Optisol-GS; Bausch & Lomb, Rochester, NY, USA) for less than 14 days before use for the experiments. These corneas were authorized for both clinical and research use by the donors’ families, and confidentiality was maintained according to the tenets of the Declaration of Helsinki.

### Isolation of Human Corneal Epithelial Cell Cultures and FGF10 Application

Human corneal epithelial cell cultures were prepared from donor corneoscleral rims. Corneoscleral donor rims were washed three times with PBS (Corning, Inc., Corning, NY, USA) and subsequently incubating in Dispase II (#D4693-1G; Sigma-Aldrich) for 1 hour at 37°C and 5% CO_2_. After incubation, cells were gently scraped from the epithelia with a surgical knife (ExactEtch, #2620K; Cytosol Ophthalmics, Lenoir, NC, USA) and plated on 12-well plates coated with 0.5 mL FNC Coating Mix (#0407; Athena Enzyme Systems, Baltimore, MD, USA) in Corneal Epithelial Cell Basal media (#PCS-700-030; American Type Culture Collection, Manassas, VA, USA) for expansion. Media were changed three times per week until cultures reached 95% confluency, at which time the cells were passaged to six-well plates and then again to 96-well plates. Passages were achieved by applying Accutase (Innovative Cell Technologies, Inc., San Diego, CA) to cultures for approximately 10 minutes to allow cells to detach and then pelleting cells at 1000 rpm for 5 minutes before resuspension in media.

When 96-well primary epithelial cell cultures reached 50% to 100% confluency, the media were changed to either the ATCC (growth-factor-containing) media or the EpiLife (growth-factor-free) media, and the cultures were tested with varying combinations of FGF-10, Gibco B-27 (#17504044; Thermo Fisher Scientific), and HCGS. FGF was tested at a concentration of 30 ng/µL. The B-27 dilutions were created by adding 2 µL of 50× stock to a 100-µL well, and HCGS dilutions were created by adding 1 µL of 100× stock to a 100-µLwell. The following experimental conditions were applied: EpiLife only (*n* = 25), EpiLife + FGF10 (*n* = 24), EpiLife + B-27 (*n* = 20), EpiLife + B-27 + FGF10 (*n* = 20), EpiLife + HCGS (*n* = 20), EpiLife + HCGS + FGF10 (*n* = 20), ATCC only (*n* = 43), and ATCC + FGF10 (*n* = 22).

Seventy-two hours after application, cell viability (proliferation) was characterized by ATP quantification utilizing the CellTiter-Glo Luminescent Cell Viability Assay (#G7570; Promega, Madison, WI, USA) and plate reader per the manufacturer's instructions. ATP quantification data collected from the plate reader were averaged and normalized to the EpiLife-only condition.

### Statistical Analysis

Statistical analyses were performed using Prism 9. First, a Shapiro–Wilk normality test was performed to evaluate whether the data followed a normal distribution. If the data passed the normality test, statistical significance between two conditions was assessed with a parametric *t*-test; otherwise, non-parametric tests were used. For results of statistical tests shown in bar graphs, the data are presented as mean ± SD of replicates from a representative experiment or of the normalized data from several experiments. Significant differences are represented as follows: ^*^P < 0.05, ^**^P < 0.01, and ^***^P < 0.001.

## Results

### FGF10 Application Increases Cell Proliferation in Adult Healthy LGs

**Figure 1.**
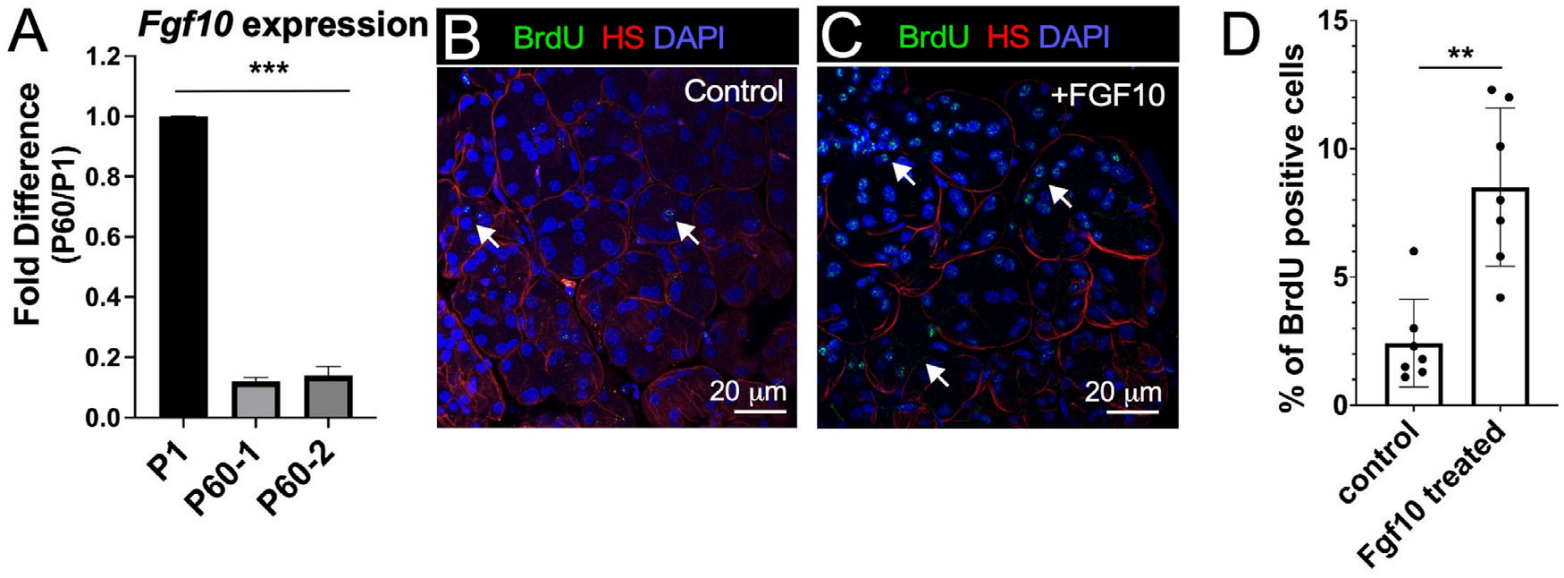
FGF10 injection increased proliferation rates. (**A**) *Fgf10* expression decreased in adult LGs, as shown by qRT-PCR using two different primer sets. (**B**, **C**) Immunostaining (BrdU in *green*, heparan sulfate [HS] in *red*, and DAPI in *blue*) of LGs injected with BSA (**B**) or with FGF10 (**C**). (**D**) Quantification of BrdU-positive cells in control (BSA-injected) and FGF10-treated LGs. One gland of each mouse was injected with BSA (control) and another gland was injected with *Fgf10* (*n* = 7; ^**^P < 0.01, nonparametric unpaired *t*-test).

The level of *Fgf10* expression, measured by quantitative reverse transcription PCR (qRT-PCR) using two sets of primers, was significantly decreased by approximately sixfold in adult LGs (at P60) compared to early postnatal LGs (at P1) (Fig. 1A). To investigate whether FGF10 modulates cell proliferation in healthy LGs, we injected the LG on one side of the wild-type *C57BL/6* mouse with FGF10 and the contralateral LG with BSA (control). Three days after this treatment, mice were injected with BrdU to assess cell proliferation in the FGF10-injected and control LGs. FGF10-injected LGs had significantly higher percentages of BrdU-positive cells than control LGs injected with BSA (Figs. 1B–1D), thus suggesting increased proliferation rate. Altogether, these findings indicate that, despite the substantial decrease in *Fgf10* expression levels after birth, adult LG cells can still respond to FGF10 stimulation by increasing replicative activity.

### FGFR2b Signaling Contributes to LG Regeneration

**Figure 2.**
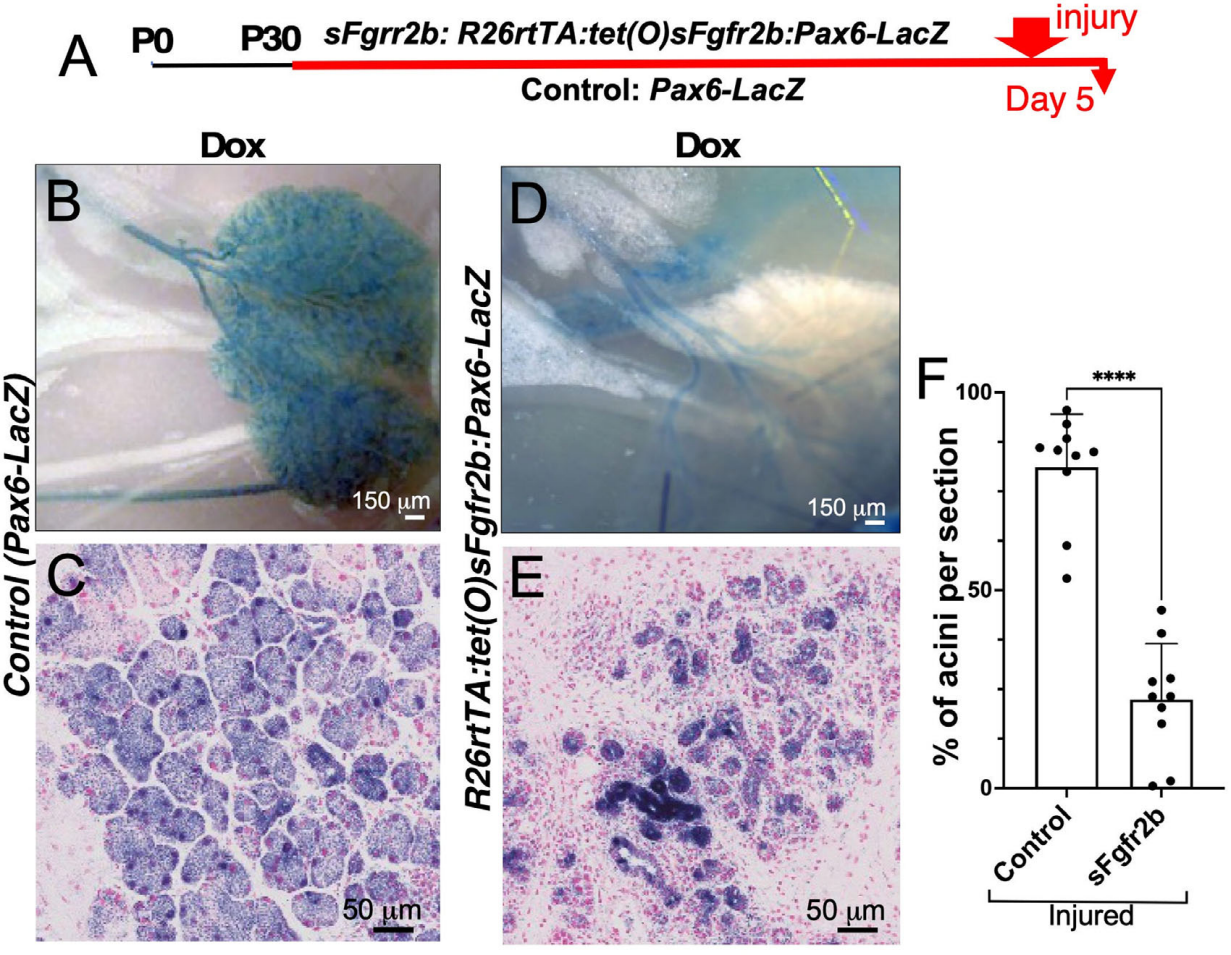
FGFR2b is necessary for LG regeneration. (**A**) To block FGFR2b signaling, we used DOX-inducible *R26rtTA:tet(O)sFgfr2b:Pax6-LacZ* transgenic mice, which, after DOX treatment, express soluble FGFR2b, attenuating normal FGFR2b function. *Pax6-LacZ* mice were used as controls. Mice received a DOX diet for 45 days, and the LGs were injured with IL-1α and analyzed five days after injury. (**B**, **C**) Injured LGs of control mice showed the presence of well-defined acinar and ductal compartments. (**D**, **E**) Injured LGs of mice expressing soluble FGFR2b had a severe reduction of the acinar compartment, but ducts were still present. (**F**) Percentage of acinar structures within the LG section was assessed by evaluation of the area represented by acini compared to the total area of the whole LG section. Five mice (10 LGs) per each group were used. ^****^P < 0.0001 using nonparametric unpaired *t*-tests (Mann–Whitney).

To assess the role of FGF2b in LG regeneration, soluble FGFR2b expression was induced in the *R26rtTA:tet(O)sFgfr2b:Pax6-LacZ* mouse model by DOX treatment. The soluble form of FGFR2b attenuates normal FGF/FGFR2b signaling. We first examined uninjured LGs of *R26rtTA:tet(O)sFgfr2b:Pax6-LacZ* mice expressing soluble FGFR2b after DOX treatment and found no defects in LG structure (not shown). To determine whether perturbation of FGFR2b signaling affects LG regeneration, we then analyzed LGs obtained from experimental and control mice (both treated with DOX) 5 days after IL-1α injury (Fig. 2A). LGs from injured control mice (*Pax6-LacZ* treated with DOX) showed the presence of acinar structures within the injected lobules, suggesting that regeneration of these glands was largely complete (Figs. 2B, 2C). In contrast, LGs from injured *R26rtTA:tet(O)sFgfr2b:Pax6-LacZ* mice showed a severe reduction of acinar structures, but ductal structures were still present (Figs. 2D, 2E). We also assessed the percentage of acinar structures within the whole LG section by quantification of the areas represented by acini compared to total area (100%) of the whole LG section. LGs of WT mice untreated with DOX and uninjured with the IL-1α were used as an additional control (Fig. 2F).

### The Level of *Fgf10* mRNA Expression Is Downregulated During LG Chronic Inflammation

**Figure 3.**
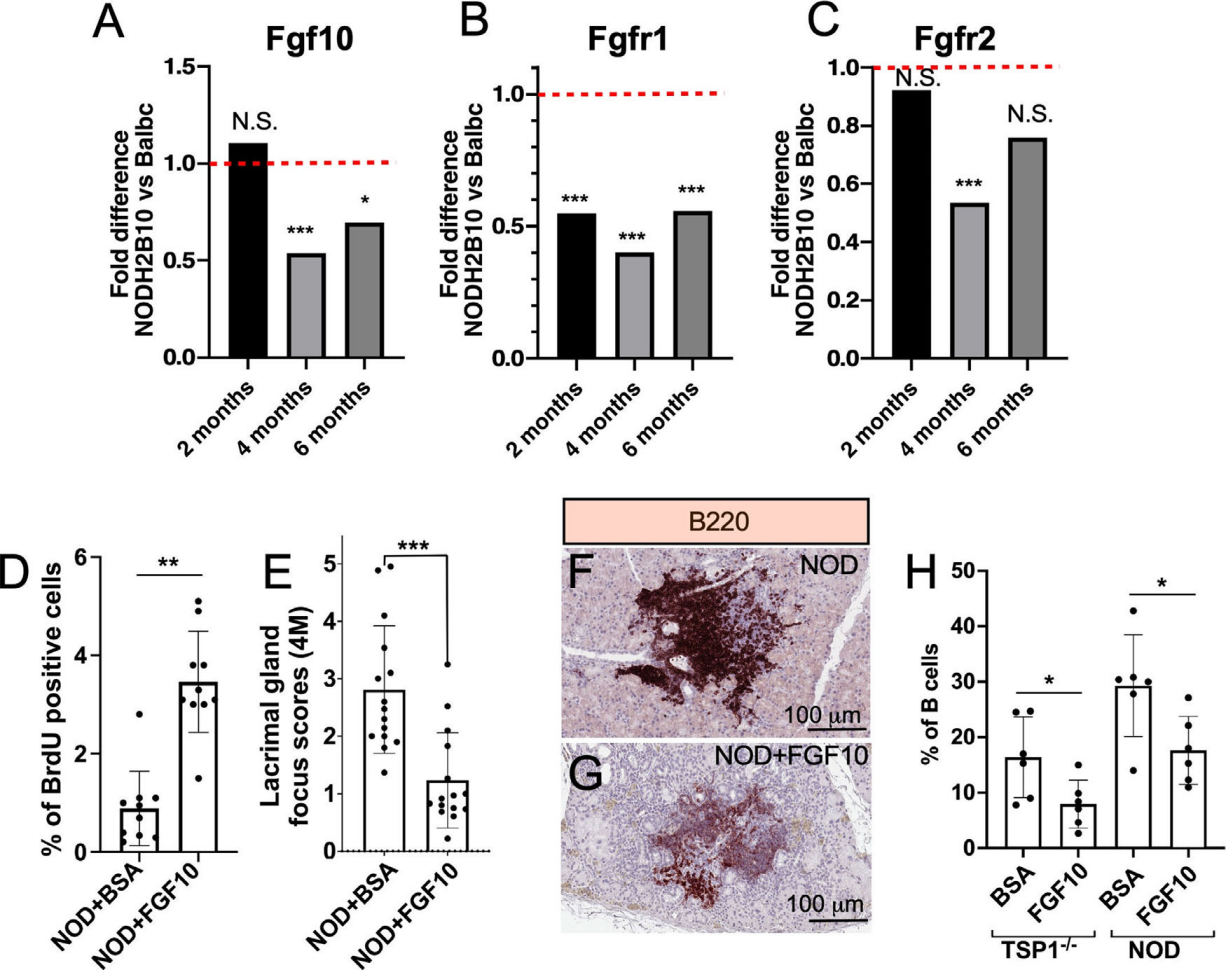
FGF10 injection into the LGs increased cell proliferation and reduced LG inflammation in the mouse models of pSS, the *NOD.B10.H2^b^* and *TSP-1*^–/–^ mice. (**A**–**C**) mRNA expression fold changes of *Fgf10* ligand (**A**) and its receptors *Fgfr1* (**B**) and *Fgfr2* (**C**) in *NOD.B10.H2^b^* mice compared to *BALB/c* at 2, 4, and 6 months of age with adjusted *P* values (FDR). (**D**) One gland of each mouse was injected with BSA (control) and another gland was injected with FGF10. Injection of FGF10 increased BrdU incorporation (*n* = 10; ^**^P < 0.01, non-parametric paired *t*-test [Wilcoxon]). (**E**) Injection of FGF10 decreased inflammation (focus scores) in the LGs of the *NOD.B10.H2^b^* mice (NOD, 4-month-old males). Three slides were analyzed for each gland (*n* = 5; ***P < 0.001, non-parametric unpaired *t*-test [Mann–Whitney]). (**F**–**H**) FGF10 injections into LGs of *TSP-1*^–/–^ and *NOD.B10.H2^b^* mice reduced B-cell infiltration as shown by immunostaining with B220 antibody (**F**, **G**; five mice per group) and by flow cytometry using CD19 antibody (**H**). In the flow cytometry experiments, one gland of each mouse was injected with BSA (control) and another gland was injected with FGF10 (*n* = 6; *P < 0.05, parametric paired *t-*test).

To determine whether the *Fgf10* expression level is altered in chronic LG inflammation, we mined our recently published bulk RNA sequencing data (GSE210332). In this dataset, LGs from the primary Sjögren's syndrome mouse model (pSS), the *NOD.B10.H2^b^* and control *BALB/c* mice, were sequenced in triplicates at 2, 4, and 6 months of age. *Fgf10* expression was significantly reduced by around twofold in *NOD.B10.H2^b^* as compared to *BALB/c* mice, specifically at the 4-month and 6-month timepoints (Fig. 3A, Table). Expression levels of other FGFs were also compared between *NOD.B10.H2^b^* mice and *BALB/c* controls, revealing a significant increase in the expression of *Fgf2* (at 6 months) and *Fgf13* (at 4 and 6 months), but no significant difference in the expression of *Fgf1, Fgf7, Fgf9, Fgf11, Fgf12, Fgf16, Fgf17, Fgf18, Fgf20*, or *Fgf22* at any age (Table). The expression of *Fgf3, Fgf4, Fgf5, Fgf6, Fgf8, Fgf14, Fgf15, Fgf21*, and *Fgf23* was not detected. It is known that *Fgf10* can signal through both *Fgfr1* and *Fgfr2*. Both *Fgfr1* and *Fgfr2* levels were significantly reduced in *NOD.B10.H2^b^* as compared to *BALB/c* mice at 4 months (Figs. 3B, 3C, Table). We also found a decrease in the expression of *Fgfr3*, but the expression of the *Fgfr4* was not affected (Table).

**Table.** Fold Change Expression of *Fgf* Family Members and Partners in *NOD* Mice Compared to *BALB/c* at 2, 4, and 6 Months of Age With Adjusted *P* Values (FDR)

	2 Months		4 Months		6 Months	
Gene	Fold Change	FDR	Fold Change	FDR	Fold Change	FDR
*Fgf1*	0.98	9.70E-01	1.17	4.12E-01	0.81	1.45E-01
*Fgf2*	1.40	3.39E-01	1.05	9.43E-01	1.99	**6.20E-04**
*Fgf3*	NE	—	NE	—	NE	—
*Fgf4*	NE	—	NE	—	NE	—
*Fgf5*	NE	—	NE	—	NE	—
*Fgf6*	NE	—	NE	—	NE	—
*Fgf7*	1.05	9.33E-01	0.78	4.95E-01	1.51	1.64E-01
*Fgf8*	NE	—	NE	—	NE	—
*Fgf9*	1.28	7.07E-01	0.95	9.76E-01	3.12	2.20E-01
*Fgf10*	1.10	8.21E-01	0.54	**4.25E-04**	0.69	**3.64E-02**
*Fgf11*	0.84	8.34E-01	0.67	4.12E-01	0.91	9.07E-01
*Fgf12*	1.00	9.97E-01	0.74	9.03E-01	0.80	9.07E-01
*Fgf13*	0.96	9.56E-01	4.48	**4.76E-03**	3.77	**3.22E-03**
*Fgf14*	NE	—	NE	—	NE	—
*Fgf15*	NE	—	NE	—	NE	—
*Fgf16*	0.87	7.24E-01	1.12	9.24E-01	0.79	9.07E-01
*Fgf17*	0.94	9.33E-01	0.69	8.40E-01	0.99	9.95E-01
*Fgf18*	0.92	9.33E-01	0.98	9.93E-01	2.29	4.67E-01
*Fgf20*	1.13	9.13E-01	0.81	9.20E-01	1.45	8.61E-01
*Fgf21*	NE	—	NE	—	NE	—
*Fgf22*	0.99	9.80E-01	1.10	9.20E-01	1.14	9.07E-01
*Fgf23*	NE	—	NE	—	NE	—
*Fgfr1*	0.55	**3.90E-19**	0.40	**9.55E-24**	0.56	**1.28E-13**
*Fgfr2*	0.92	7.28E-01	0.53	**1.03E-07**	0.76	7.15E-02
*Fgfr3*	0.62	**4.93E-02**	0.41	**1.34E-02**	0.88	8.78E-01
*Fgfr4*	1.03	9.33E-01	0.90	9.20E-01	1.52	7.83E-01
*Kl*	−0.05	9.33E-01	0.28	9.03E-01	0.33	9.07E-01

NE, not expressed.

Bold type indicates FDR < 0.05.

### Injection of FGF10 into The LG Reduces Inflammation and Improves Repair During Chronic Inflammation

Similar to healthy LGs of WT mice (see Fig. 1), injection of FGF10 into the LGs of *NOD.B10.H2^b^* mice increased cell proliferation as measured by the percentage of BrdU-positive cells (1%) compared to control (3.5%) (Fig. 3D). Additionally, the degree of inflammation in the LGs could be assessed by the focus score. We observed a significant decrease in immune cell infiltration in the LGs injected with FGF10 (focus score = 1) as compared to *NOD.B10.H2^b^* glands injected with BSA (focus score = 3) (Fig. 3E).

Chronic inflammation is characterized by increased focal infiltration of T and B cells with a prevalence of B cells in humans with SS and in SS mouse models.^43^*^,^*^44^ At 4 months, the LGs of *NOD.B10.H2^b^* males showed numerous lymphocytic infiltrations with a large number of B cells.^45^ Thus, we determined the proportion of B cells in the LG of the *TSP-1*^–/–^ and *NOD.B10.H2^b^* mice to evaluate the level of inflammation in the LG. To determine how FGF10 application affects lymphocytic foci, we generated paraffin sections of control BSA-injected *NOD.B10.H2^b^* (Fig. 3F) and FGF10-injected (Fig. 3G) *NOD.B10.H2^b^* LGs and immunostained them with the CD45R (B220, B-cell marker) antibody. We found a decrease in B-cell labeling within the LG infiltration foci of the FGF10-treated glands (Fig. 3G). Next, we performed quantification of B cells using flow cytometry in LGs from both *NOD.B10.H2^b^* and *TSP-1*^–/–^ mice injected with FGF10 or BSA control. We found that FGF10 injection into LGs significantly decreased the number of B cells in the LGs of both SS mouse models, *NOD.B10.H2^b^* and *TSP-1*^−/−^ (Fig. 3H), whereas control LGs of 4-month-old WT *C57BL/6* and *BALB/c* mice showed very small numbers of B cells (not shown).

### FGF10 Increases Cell Viability of Mouse and Human Primary Corneal Epithelial Cultures

**Figure 4.**
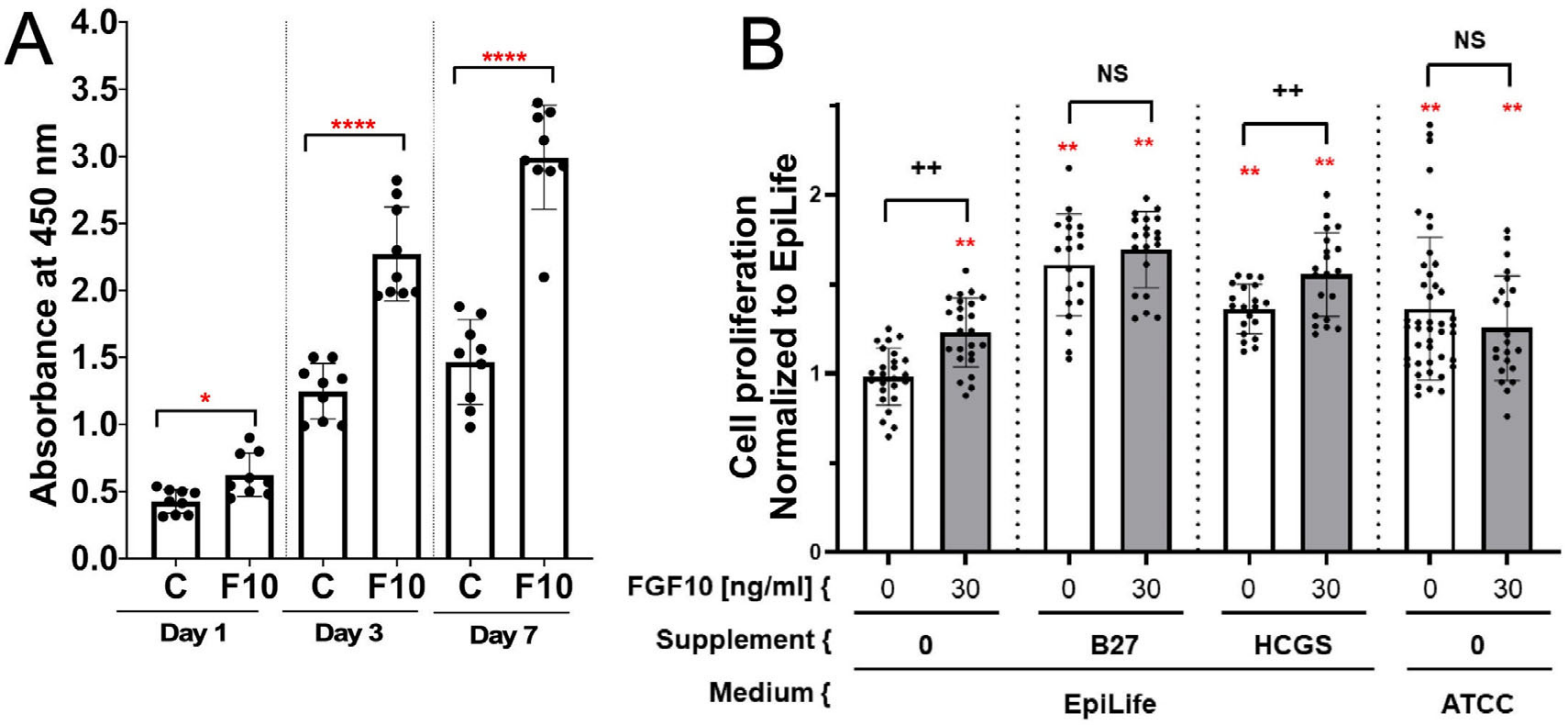
FGF10 increased the viability of primary mouse corneal epithelial cells. (**A**) Mouse corneal epithelial cells were seeded in multi-well dishes, treated with FGF10 (30 ng/mL) or BSA (control), and cultured for 1, 3, and 7 days. Cells were processed for viability assay (tetrazolium salt, WST- 8). Dots represent the number of repeats (*n* = 9) for each condition/time. Statistical significance was assessed with unpaired *t*-tests. All *bar plots* are mean ± SD. ^*^P < 0.05; ^****^P < 0.0001. (**B**) FGF10, B27, and HCGS improved cell viability in human corneal epithelial cell cultures. The graph shows normalized raw data, means, and standard deviations for each corneal epithelial cell culture condition using the ATP quantification cell viability assay. ^**^P < 0.01 compared to EpiLife without supplements control; ^++^*P* < 0.01; NS, not significant compared to the control with the same supplement but without *Fgf10*.

To determine whether FGF10 can modulate primary mouse corneal cell proliferation, the viability of mouse corneal epithelial cells with and without FGF10 was analyzed by WST-8 assay (Fig. 4A). Cell viability increased significantly in cultures treated with FGF10 compared to control cultures at all time points (^*^P < 0.05). Similar studies were performed using human corneal epithelial cells cultured in various defined media with and without FGF10 (Fig. 4B). The addition of either B-27 or HCGS to the EpiLife media caused a significant increase in the number of viable cells (61% ± 28% and 36% ± 14%, respectively; *P* < 0.01) compared to the media alone. The addition of FGF10 increased cell viability compared to EpiLife media alone (23% ± 19%; *P* < 0.01). In addition to the individual pro-survival effects of B-27, HCGS, and FGF10 alone, we found that the combination of FGF10 with B-27 or FGF10 with HCGS in EpiLife media resulted in a further significant increase in corneal cell viability compared to EpiLife alone (69% ± 21% and 55% ± 23%, respectively; *P* < 0.01), as well as compared to EpiLife with only B-27 or HCGS (*P* < 0.01). We also cultured cells in ATCC media with and without FGF10. The ATCC media alone sustained greater viability than the EpiLife media (36% ± 4%; *P* < 0.01); however, the addition of FGF10 to ATCC media did not increase the number of viable cells compared to ATCC media alone.

## Discussion

Several studies have shown that FGF10 is required for human and mouse LG development.^13^^,^^16^^,^^19^^,^^46^ In this study, we have shown that FGF10 application in vitro and in vivo significantly increases the viability and proliferation of LG epithelial cells and decreases the number of infiltrating cells in an injured gland. We have recently shown that cell proliferation rate in the exocrine LG decreases during postnatal development coincident with LG maturation.^47^ Our current finding that *Fgf10* expression is decreased in adult LGs versus early postnatal LGs could help to explain the decrease in cell proliferation in adult LGs and supports the idea that the adult LG is a stable structure with a low rate of cellular turnover.

Despite this low cellular turnover under homeostatic conditions, the healthy LG has a high regenerative potential and is able to repair itself even after substantial damage^37^; however, this ability decreases with chronic inflammation.^48^^,^^49^ Our RNA-sequencing data showed that *Fgf10* expression in adult glands decreases with progression of inflammation. This suggests that loss of FGF10 may be involved in the loss of regenerative capacity associated with chronic inflammation. Moreover, FGFR2b signaling is also required for lacrimal and submandibular gland development.^16^^,^^18^^,^^50^ Of note, previous ablation of FGFR2 in keratin 14 (K14)-expressing adult cells using the *K14rtTA-tetOCre-Fgfr2^fl^*^/^*^fl^* mouse led only to meibomian gland atrophy and did not affect the LG, suggesting that FGFR2 is not required for adult LG homeostasis. However, interpretation of this study was complicated by the fact that, although K14 is expressed throughout the whole gland epithelium during development,^51^ in adult LGs K14 is found only in the basal ductal and myoepithelial cells.^47^ Our experiments allowed perturbation of FGFR2b signaling in all LG cells expressing the receptor; hence, our finding that uninjured LGs do not require FGFR2 expression is important confirmation of the idea that the adult LG is a stable structure with a low rate of cell turnover. It remains possible that long-term perturbation of the FGFR2b could produce gland atrophy. Importantly, our finding of a pronounced delay in adult gland regeneration after genetic perturbation of FGFR2b indicates that FGF10/FGFR2b signaling is a non-redundant regulator of gland repair, likely by recapitulating its developmental functions in driving cell proliferation and branching morphogenesis. Our histological findings showing a severe reduction in acinar structures compared to ductal structures in these tissues further suggest that regeneration of the ductal epithelium may be less dependent on the FGFR2b signaling than the acinar epithelium.

Our finding that FGF10 injections into LGs also reduced immune cell infiltration suggests that it might mitigate the inflammatory response that is often characteristic of dry eye. Moreover, our data also suggest that FGF10 injections can accelerate repair processes in chronically inflamed glands. However, it is important to emphasize that this study did not focus on inflammatory processes, and further studies would be necessary to understand the precise role of FGF10 in modulating inflammation.

This study has some limitations. First, we used a small sample size (three mice per group per age) for bulk RNA sequencing analysis. Still, in our previous study, we found that lymphocytic infiltrates consistently appeared in the *NOD.B10.H2^b^* males at 2 months and progressed into the disease at 4 to 6 months.^52^ We also used only male *NOD.B10.H2^b^* mice for RNA sequencing and other experiments. Male *NOD.B10.H2^b^* mice develop severe LG inflammation within the range of 2 to 6 months, but females of this mouse model develop the disease much later (4–12 months). Thus, analysis of males allowed us to study the expression of FGF genes and their receptors in adult mice free of aging-related changes.

We have not explored the effect of FGF10 on the proliferation of corneal epithelial cells during chronic inflammation in vivo because the efficiency of FGF10 in promoting corneal epithelial healing in the rabbit model of dry eye has been previously evaluated.^53^ Additionally, we used the proportion of B cells and focus scores to evaluate the level of inflammation in the LG. Although analyzing the effect of *Fgf10* on the different immune cell populations would be of great interest, the extensive study of resident and infiltrating immune cells is beyond the scope of this study.

Our results also show that exogenous FGF10 can increase cell proliferation of in vitro primary corneal epithelial cultures and can provide synergistic proliferative effects when applied in combination with other growth factors. This finding might be exploited for tissue engineering purposes, such as the development of corneal or LG grafts. Altogether, our findings on the role of FGF10/FGFR2b signaling in adult LG regeneration have significant clinical implications for FGF10 as a potential treatment of dry eye disease that may target underlying mechanisms of inflammation and decreased cellular regeneration and proliferation.
